# Reviewer acknowledgement 2015

**DOI:** 10.1186/s13601-016-0104-0

**Published:** 2016-04-06

**Authors:** Clive Grattan

**Affiliations:** Norfolk and Norwich University Hospital, Norwich, UK

The Editor of *Clinical and Translational Allergy* would like to thank all of the reviewers who contributed to the journal in Volume 5 (2015).

**Ioana Agache**

Romania

**Jaap Akkerdaas**

Netherlands

**Alberto Alvarez-Perea**

Spain

**Elisabeth Angiers**

UK

**Dario Antolin-Amerigo**

Spain

**Michael Arden Jones**

UK

**Riccardo Asero**

Italy

**Maria Basagaña**

Spain

**Sevim Bavbek**

Turkey

**Cecilia Berin**

USA

**Leif Bjermer**

Sweden

**Barbara Bohle**

Austria

**Elisa Boni**

Italy

**Elena Borzova**

UK

**Helen Brough**

USA

**Indre Butiene**

Lithuania

**Betul Buyuktiryaki**

USA

**Luis Caraballo**

Colombia

**Victoria Cardona**

Spain

**Ozlem Cavkaytar**

Turkey

**Lorenzo Cecchi**

Italy

**Yoon-Seok Chang**

South Korea

**Cemal Cingi**

Turkey

**Merce Corominas**

Spain

**Alfonso Cuvillo**

Spain

**Eduard Diogene**

Spain

**Javier Domínguez**

Spain

**Maria Teresa Dordal Culla**

Spain

**Motohiro Ebisawa**

Japan

**Ibon Eguiluz Gracia**

Norway

**Philippe Eigenmann**

Switzerland

**Peter Andreas Eng**

Switzerland

**Filippo Fassio**

Italy

**Marta Ferrer**

Spain

**Penny Fitzharris**

New Zealand

**Joao Fonseca**

Portugal

**Vanessa Garcia Larsen**

UK

**Roy Gerth van Wijk**

Netherlands

**Rolla Giovanni**

Italy

**Nikolaos Gkavogiannakis**

Greece

**Maximiliano Gomez**

Argentina

**Francisca Gómez**

Spain

**Enrico Heffler**

Italy

**Dolores Hernandez**

Spain

**Valerie Hox**

Belgium

**Louisa James**

USA

**Holloway John**

UK

**Manel Jordana**

Canada

**Marek Jutel**

Poland

**Omer Kalayci**

Turkey

**Livje Kalogjera**

Croatia

**Edward Knol**

Netherlands

**Anastasios Konstantinopoulos**

Greece

**Marcin Kurowski**

Poland

**Désirée Larenas Linnemann**

Mexico

**Heung-Man Lee**

South Korea

**Bo Liu**

USA

**Ramon Lleonart**

Spain

**Olga Luengo**

Spain

**David Luyt**

UK

**Nadine Marrouche**

UK

**Marcus Maurer**

Germany

**Ervin Mingomataj**

Albania

**Dimitris Mitsias**

USA

**Mario Morais-Almeida**

Portugal

**Carmen Moreno**

Spain

**Dilsad Mungan**

Turkey

**Antonio Nieto**

Spain

**Bright Nwaru**

UK

**Nerin Onder**

Cyprus

**Öner Özdemir**

Turkey

**Punchama Pacharn**

Thailand

**Giovanni Pajno**

Italy

**Nikolaos Papadopoulos**

Greece

**Alexandra Papadopoulou**

Greece

**Giovanni Passalacqua**

Italy

**Lars K. Poulsen**

Denmark

**Constantinos Ptsios**

Greece

**Santiago Quirce**

Spain

**Georgios Rentzos**

Sweden

**Graham Roberts**

UK

**Cansin Sackesen**

Turkey

**Sejal Saglani**

UK

**Umit Sahiner**

Turkey

**Rohit Saluja**

Germany

**Mario Sanchez-Borges**

Venezuela

**Conceicao Santos**

Portugal

**Joaquin Sastre**

Spain

**Guy Scading**

USA

**Bulent Sekerel**

Turkey

**Mohamed Shamji**

UK

**Aziz Sheikh**

UK

**Helen Smith**

UK

**Woo-Jung Song**

South Korea

**P. Sripalakit**

Thailand

**Miguel Angel Tejedor**

Spain

**Olympia Tsilochristou**

Greece

**Victor Turcanu**

UK

**Ozge Uysal Soyer**

Turkey

**Antonio Valero**

Spain

**Eva Varga**

Austria

**Carmen Vidal**

Spain

**Berber Vlieg-Boerstra**

Netherlands

**Emmanuel Xystrakis**

UK

**S. Tolga Yavuz**

Turkey

**Mario Zernotti**

Argentina

